# A Novel Functional Site in the PB2 Subunit of Influenza A Virus Essential for Acetyl-CoA Interaction, RNA Polymerase Activity, and Viral Replication*[Fn FN2]

**DOI:** 10.1074/jbc.M114.559708

**Published:** 2014-07-25

**Authors:** Dai Hatakeyama, Masaki Shoji, Seiya Yamayoshi, Takenori Hirota, Monami Nagae, Shin Yanagisawa, Masahiro Nakano, Naho Ohmi, Takeshi Noda, Yoshihiro Kawaoka, Takashi Kuzuhara

**Affiliations:** From the ‡ Laboratory of Biochemistry, Faculty of Pharmaceutical Sciences, Tokushima Bunri University, Tokushima 770-8514, Japan,; the § Department of Microbiology and Immunology, Division of Virology, Institute of Medical Science, and; the ‖ Department of Special Pathogens, International Research Center for Infectious Diseases, Institute of Medical Science, University of Tokyo, Tokyo 108-8639, Japan,; ¶ PRESTO, Japan Science and Technology Agency, Saitama 332-0012, Japan, and; the ** Department of Pathobiological Sciences, School of Veterinary Medicine, University of Wisconsin-Madison, Madison, Wisconsin 53711

**Keywords:** Acetyl-coenzyme A (Acetyl-CoA), Influenza Virus, Negative-strand RNA Virus, Recombinant Protein Expression, RNA Polymerase

## Abstract

The PA, PB1, and PB2 subunits, components of the RNA-dependent RNA polymerase of influenza A virus, are essential for viral transcription and replication. The PB2 subunit binds to the host RNA cap (7-methylguanosine triphosphate (m^7^GTP)) and supports the endonuclease activity of PA to “snatch” the cap from host pre-mRNAs. However, the structure of PB2 is not fully understood, and the functional sites remain unknown. In this study, we describe a novel Val/Arg/Gly (VRG) site in the PB2 cap-binding domain, which is involved in interaction with acetyl-CoA found in eukaryotic histone acetyltransferases (HATs). *In vitro* experiments revealed that the recombinant PB2 cap-binding domain that includes the VRG site interacts with acetyl-CoA; moreover, it was found that this interaction could be blocked by CoA and various HAT inhibitors. Interestingly, m^7^GTP also inhibited this interaction, suggesting that the same active pocket is capable of interacting with acetyl-CoA and m^7^GTP. To elucidate the importance of the VRG site on PB2 function and viral replication, we constructed a PB2 recombinant protein and recombinant viruses including several patterns of amino acid mutations in the VRG site. Substitutions of the valine and arginine residues or of all 3 residues of the VRG site to alanine significantly reduced the binding ability of PB2 to acetyl-CoA and its RNA polymerase activity. Recombinant viruses containing the same mutations could not be replicated in cultured cells. These results indicate that the PB2 VRG sequence is a functional site that is essential for acetyl-CoA interaction, RNA polymerase activity, and viral replication.

## Teaser

**Background:** The PB2 subunit of RNA polymerase of influenza virus has a novel site for acetyl-CoA interaction, which contains a valine/arginine/glycine (VRG) motif.

**Results:** A mutation in the VRG site inhibited acetyl-CoA interaction, RNA polymerase activity, and viral replication.

**Conclusion:** Acetyl-CoA interaction at the VRG site is potentially essential for viral replication.

**Significance:** The VRG site might be a novel target for drugs and vaccines.

## Introduction

Influenza A is still considered a serious global health issue (1). The avian H5N1 influenza A virus was reported to show stronger virulence to human by single amino acid substitutions in PB2 and hemagglutinin (2), and new strains of this virus, such as the swine-originating A/H1N1 in 2009 and the avian-originating A/H7N9 in 2013, had since emerged serially (1, 3). To combat the influenza A virus, zanamivir and oseltamivir, which inhibit neuraminidase activity, are widely used as antiviral drugs (4). However, because the viral neuraminidase gene mutates easily, many strains, including A/H1N1 and A/H7N9, were found to be resistant to these drugs soon after their first discovery (5, 6). In addition, oseltamivir, the most widely prescribed anti-influenza medication, was reported to stimulate behavioral activities in adolescents (7), and we recently suggested that the inactive prodrug of oseltamivir (oseltamivir ethyl ester) inhibits the activity of monoamine oxidase A, and this side effect produced the stimulant effect (8). Therefore, the identification of novel target molecules is required to develop antiviral therapies. In particular, viral RNA-dependent RNA polymerase (RdRp),^2^ which contains the PA, PB1, and PB2 subunits (9, 10), is a promising target in the development of antiviral drugs. We and other groups succeeded in identifying several chemicals as potential anti-influenza agents, which inhibited the functions of viral RdRp (11–15).

The structure and function of the viral PB2 subunit has been well studied: its N-terminal region plays an essential role in viral RNA replication (16), the central region binds to the cap sequence (7-methylguanosine triphosphate; m^7^GTP) of the pre-mRNA of the host for “cap snatching” (17), and its C-terminal region contains a nuclear localization signal that mediates its interaction with importin (17). We and other groups have shown that lysine 627 located in the C-terminal region of the PB2 subunit is positively associated with high pathogenicity and host range restriction of the influenza A virus (18–20). Crystallography of the PB1 and PB2 complex identified the amino acid residues required for interaction of these subunits, and substitution of these amino acid residues quenched the polymerase activity (21).

Acetyl-coenzyme A (acetyl-CoA) is involved in modulating protein function. The most important role of acetyl-CoA is to work as a cofactor in the acetylation of histones (22, 23) and other proteins (24–28). Interestingly, several proteins possess autoacetyltransferase activity (29–37). Acetyl-CoA not only supplies its acetyl group to proteins, but, in doing so, also modifies the function of the target proteins. RNA polymerase II transcription is stimulated by acetyl-CoA in the absence of histones, and transcriptional stimulation results from increased affinity of the transcriptional factor IID for the DNA promoter (38). In addition, acetyl-CoA was also reported to bind to bacterial phosphoenolpyruvate carboxylase and modulate its enzymatic activity (39). These results suggest that acetyl-CoA can interact with various types of proteins and act as a cofactor that affects protein function. Notably, the specific amino acid sequence required for acetyl-CoA binding to the eukaryotic GCN5 protein, a histone acetyltransferase (HATs), has also recently been identified (40, 41).

Here, we found that the Val/Arg/Gly (VRG) site required for acetyl-CoA interaction with eukaryotic HATs was also conserved in the viral PB2 subunit cap-binding domain. We also performed a series of biochemical experiments that demonstrate the interaction with acetyl-CoA in this domain. Amino acid mutations in the VRG site eliminated the interaction with acetyl-CoA, decreased RNA polymerase activity of viral RdRp, and inhibited viral growth. This is the first report to identify a new PB2 subunit functional site that is required for replication of the influenza virus.

## EXPERIMENTAL PROCEDURES

### 

#### 

##### Screening of Three-dimensional Protein Structures Similar to the PB2 Cap-binding Domain

Tertiary structures similar to the PB2 cap-binding domain (PDB code 2vqz) were screened in a three-dimensional protein structure library using the online Markovian Transition of Structure (MATRAS) evolution program (42).

##### Expression and Purification of the PB2 Cap-binding Domain Subunit

The new region in the recombinant protein was from amino acids 322 to 783, which covers the cap-binding domain (GenBank^TM^ Accession number AAO46493) of the PB2 polymerase subunit (influenza A virus (A/Hong Kong/16/1968(H3N2))). Wild-type or mutated PB2 cap-binding domain subunit was amplified by PCR using the primers listed below. To amplify mutant PB2 at each lysine residue or the VRG motif, an overlap extension method was performed for: PB2-WT-F, TTT TTT TGC TAG CTC CTT CAG TTT TGG CGG ATT C (the restriction site for NheI); PB2-WT-R, AAG GAT CCT TAC ATT TTG CTG ACT CTT ATC CCT C (the restriction site for BamHI); PB2-Mut-K331A-F, GCT AGC TCC TTC AGT TTT GGC GGA TTC ACA TTT GCG AGA ACA AGC GGG (the restriction site for NheI); PB2-Mut-K339A-OLE-F, GGG TCA TCA ATC GCG AGA GAG GAA GAA TTG; PB2-Mut-K339A-OLE-R, CGC GAT TGA TGA CCC GCT TGT TCT CTT AAA TGT GAA TCC GCC; PB2-Mut-K353A-OLE-F, CTC CAA ACA TTA GCA ATA AGG GTG CAT G; PB2-Mut-K353A-OLE-R, CAT GCA CCC TTA TTG CTA ATG TTT GGA G; PB2-Mut-K368A-OLE-F, ACA ATG GTG GGG GCA AGG GCA ACA GCT; PB2-Mut-K368A-OLE-R, GC TGT TGC CCT TGC CCC CAC CAT TGT; PB2-Mut-K376A-OLE-F, GCT ATA CTC AGA GCA GCA ACC AGG AGA TTG; PB2-Mut-K376A -OLE-R, CAA TCT CCT GGT TGC TGC TCT GAG TAT AGC; PB2-Mut-K412A-OLE-F, GAA GAT TGC ATG ATA GCA GCA GTT AGA GGT G; PB2-Mut-K412A-OLE-R, CAC CTC TAA CTG CTG CTA TCA TGC AAT CTT C; PB2-Mut-K440A-OLE-F, AGG CAT TTT CAG GCA GAT GCG AAA GTG; PB2-Mut-K440A-OLE-R, CAC TTT CGC ATC TGC CTG AAA ATG CCT; PB2-Mut-K443A-OLE-F, CAG AAA GAT GCG GCA GTG CTT TTT CAA; PB2-Mut-K443A -OLE-R, TTG AAA AAG CAC TGC CGC ATC TTT CTG; PB2-Mut-K482A-R, GGA TCC TTA CAT TGC GCT GAC TCT TAT CCC TCT C (the restriction site for BamHI); PB2-VRG-mut1-F, GCA TGA TAA AAG CAG CTA GAG GTG ATC TGA ATT TCG; PB2-VRG-mut1-R, GCT TTA AGT CTA GTG GAG ATC GAC GAA AAT AGT ACG; PB2-VRG-mut2-F, GCA TGA TAA AAG CAG TTG CAG GTG ATC TGA ATT TCG; PB2-VRG-mut2-R, CGA AAT TCA GAT CAC CTG CAA CTG CTT TTA TCA TGC; PB2-VRG-mut3-F, GCA TGA TAA AAG CAG TTA GAG CTG ATC TGA ATT TCG; PB2-VRG-mut3-R, CGA AAT TCA GAT CAG CTC TAA CTG CTT TTA TCA TGC; PB2-VRG-mut4-F, GCA TGA TAA AAG CAG CTG CAG GTG ATC TGA ATT TCG; PB2-VRG-mut4-R, CGA AAT TCA GAT CAC CTG CAG CTG CTT TTA TCA TGC; PB2-VRG-mut5-F, GCA TGA TAA AAG CAG TTG CAG CTG ATC TGA ATT TCG; PB2-VRG-mut5-R, CGA AAT TCA GAT CAG CTG CAA CTG CTT TTA TCA TGC; PB2-VRG-mut6-F, GCA TGA TAA AAG CAG CTA GAG CTG ATC TGA ATT TCG; PB2-VRG-mut6-R, CGA AAT TCA GAT CAG CTC TAG CTG CTT TTA TCA TGC; PB2-VRG-mut7-F, GCA TGA TAA AAG CAG CTG CAG CTG ATC TGA ATT TCG; PB2-VRG-mut7-R, CGA AAT TCA GAT CAG CTG CAG CTG CTT TTA TCA TGC.

Each PCR product was ligated into the pET28a(+) vector (Novagen). This construct was then transformed into BL21-CodonPlus *Escherichia coli* cells (Stratagene). His_6_-tagged recombinant protein expression was induced by treatment with 0.32 mm isopropyl β-d-thiogalactopyranoside in TBG-M9 medium (0.8% Bacto-tryptone (BD Bioscience) and 0.4% NaCl), followed by purification using nickel-nitrilotriacetic acid-agarose (Qiagen). His_6_-tagged proteins were purified using a HiTrap carboxymethyl-FF column (GE Healthcare) and the ÄKTAprime plus system (GE Healthcare). The recombinant protein containing the PA N-terminal domain was produced as described previously (12–14).

##### Binding Assays Using Radioisotope-labeled Acetyl-CoA

The procedures used to perform this study were modified from a previous report (43). Recombinant PB2 cap-binding domain proteins (1 μg) were incubated with 18.5 kBq of [^3^H]acetyl-coenzyme A or [acetyl-^3^H]acetyl-coenzyme A (1.2 μm; 233 mCi/mmol; MT-896H, Moravek Biochemicals) or [^14^C]acetyl-CoA or [acetyl-1-^14^C]acetyl-coenzyme A (112 μm; 2 mCi/mmol; NEC-313, PerkinElmer Life Sciences) at 30 °C for 30 min in buffer containing 50 mm Tris-HCl (pH 8.0), 10% glycerol, 1 mm dithiothreitol, and 10 mm sodium butyrate. To measure the radioactivity of [^3^H], the reaction was filtered on a cation exchange filter (P81; Whatman). Filter papers were washed in a 0.2 m sodium carbonate solution (pH 9.2) twice for 30 min each, dried in a vacuum evaporator, and quantified in a liquid scintillation counter (LSC-6100; ALOKA). To detect ^14^C radioactivity, reactions were mixed with 2× SDS loading dye, boiled for 2 min, and separated in 14% SDS-PAGE gels, after which an imaging plate was exposed to such a gel for several days. Signals were detected using a fluoroimage analyzer (FLA-2000; Fuji Film). In the competitive binding experiment with m^7^GTP, 1 μg of recombinant PB2 cap-binding domain proteins was incubated with various concentrations of m^7^GTP for 30 min at 30 °C. Curcumin (Sigma), anacardic acid (Sigma), and garcinol (Enzo Life Sciences) were prepared in dimethyl sulfoxide (DMSO). CoA (Oriental Yeast), cap structure (7-methylguanosine 5′-diphosphate, m^7^GTP, Sigma), epigallocatechin-3-gallate (Sigma), and plumbagin (Tokyo Chemical Industry) were prepared in distilled water. These chemicals were mixed in the reaction solution prior to incubation, and incubated for 30 min at 30 °C after the addition of 1 μg of recombinant PB2 cap-binding domain proteins.

##### Anti-influenza Activity of HAT Inhibitors

Cultured Madin-Darby canine kidney (MDCK) cells were seeded and grown in each well of a 24-well plate. The cells were infected with influenza A/PR/8/34 virus at the multiplicity of infection of 0.001 in an infectious medium (DMEM supplemented with 1% bovine serum albumin, 1× penicillin-streptomycin (Invitrogen) and 4 mm l-glutamine, and incubated for 1 h at 37 °C under 5% CO_2_. After infection, unbound viruses in the medium were removed and then the cells were treated with DMSO (0.5%), ribavirin (50 μm, WAKO), anacardic acid (50 μm), or garcinol (50 μm) in the infectious medium supplemented with 3 μg/ml of TPCK-treated trypsin (Sigma) at 37 °C in a humidified atmosphere of 5% CO_2_. After 24, 48, or 72 h, supernatants were collected from each well. Viral titers were determined using immunostaining (44). Briefly, the supernatants were serially diluted and then added to the culture medium of MDCK cells. The cells were incubated with this mixture for 16 h at 37 °C in a humidified atmosphere of 5% CO_2_. Thereafter, the cells were fixed with 4% paraformaldehyde in phosphate-buffered saline (−) for 30 min at 4 °C and then permeabilized with 0.3% Triton X-100 for 20 min at room temperature. A mouse anti-influenza A nucleoprotein (NP) antibody (FluA-NP 4F1; Southern Biotech) and horseradish peroxidase-conjugated goat anti-mouse IgG antibody (SouthernBiotech) were used as primary and secondary antibodies, respectively. To visualize the infected cells, TrueBlue peroxidase substrate (KPL, Inc.) was added, and color development was terminated after 15 min by washing with H_2_O. The stained cells were counted under a microscope, and then viral titers were calculated.

##### In Silico Docking Simulation Analysis of Acetyl-CoA and the Influenza RNA Polymerase PB2 Cap-binding Domain

Molecular modeling was performed using the Molecular Operating Environment software (MOE; Chemical Computing Group, Quebec, Canada). The x-ray crystallographic structure of the cap-binding domain of PB2 (PDB code 2vqz) was obtained from the Protein Data Bank. To prepare the enzyme for docking studies: (i) the ligand molecule was removed from the active site of the enzyme; (ii) hydrogen atoms were added to the structure using standard geometry; (iii) the structure was minimized using an MMFF94x force-field; (iv) MOE Alpha Site Finder was used for active site searches within the enzyme structure and dummy atoms were created from the obtained α spheres; and (v) the obtained model was then applied to the Dock program (Ryoka Systems Inc., Tokyo, Japan). The acetyl-CoA conformations were generated by a stochastic search in MOE and docking simulations with PB2 were undertaken for each of these conformations. The rescoring steps (1 and 2) were performed using the Affinity dG program with the retain parameter set at 30. Refinement was performed using the Forcefield program.

##### Binding Assays with m^7^GTP Immobilized on Agarose Resin

One μg of the wild-type and mutant recombinant PB2 were incubated at 30 °C for 30 min in buffer consisting of 50 mm Tris-HCl (pH 8.0), 10% glycerol, 1 mm dithiothreitol, and 10 mm sodium butyrate. After centrifugation at 20,000 × *g* for 20 min to remove the precipitate, immobilized γ-aminophenyl-m^7^GTP linked via a C10-spacer (AC-155, Jena Bioscience) was added to the supernatants and incubated at 4 °C overnight with gentle rotation. Samples of resins and supernatants were separated in 14% SDS-PAGE gels and visualized using Coomassie Brilliant Blue staining.

##### In Vitro Transcription Assay

The transcription level of purified ribonucleoprotein (RNP) was measured by primer extension. The procedures of primer extension analyses were performed as described previously (45), with modifications. Nucleotide sequences of neuraminidase gene-specific primers were the same as those used in Ref. 45. Prior to the primer extension analyses, the 5′ end of each primer (10 pmol) was phosphorylated using 20 pmol of [γ-^32^P]ATP (PerkinElmer Life Sciences) using the enclosed protocol of the DNA 5′-End Labeling System (Promega). Each primer was purified using the Mini Quick Spin Oligo Columns Sephadex G-25 (Roche Applied Science). The RNP complex was purified from virions of A/Puerto Rico/8/34 (PR8, H1N1) as described previously (46). Purified RNP (0.5 μg) was incubated with 1 mm ATP, 0.5 mm CTP, 0.5 mm GTP, 0.5 mm UTP, 5 mm MgCl_2_, 1 mm dithiothreitol (DTT), 1 unit of RNaseOUT^TM^ Recombinant Ribonuclease Inhibitor (Invitrogen), and different concentrations of acetyl-CoA, with 0.03 μg of rabbit globin mRNA (Sigma) at 30 °C overnight, in a 4-μl reaction. After extraction with the RNeasy Mini Kit (Qiagen), the RNA samples were mixed with 0.1 pmol of the ^32^P-labeled primer in a 5-μl reaction, and incubated at 95 °C for 5 min and 45 °C for 1 min. Five microliters of 2× transcription mixture (50 units of SuperScript II Reverse Transcriptase or SuperScript III Reverse Transcriptase, 2× first-strand buffer (all from Invitrogen), 20 mm DTT, and 1 mm dNTP mixture) was added to the mixture of an RNA sample and the ^32^P-labeled primer and incubated at 37 °C for 90 min. The reaction was stopped by addition of 8 μl of 90% formamide and 10 mm EDTA and heating at 95 °C for 3 min. Proteins in the samples were separated by electrophoresis in a 6% denatured polyacrylamide gel containing 7 m urea and Tris borate-EDTA (TBE) buffer, dried on filter papers, and detected by autoradiography.

##### Strand-specific qRT-PCR

A549 cells were seeded and grown in each well of a 24-well plate. The cells were transfected with pCA-NP, PA, PB1, and PB2 or its mutants (0.2 μg each), and with pPolI-NA (segment 6, 0.1 μg). After 24 h, total RNA was extracted from each transfectant clone using RNeasy Mini Kit (Qiagen). We chose segment 6 for assessing the RNA level. Reverse transcription was performed as described previously for synthesis of cDNA from each strand of vRNA, cRNA, and mRNA using primers of the same nucleotide sequence (47). We also used a random hexamer for reverse transcription, which was used for qRT-PCR of 18 S rRNA as an internal control. Real-time PCR was performed using SYBR® Green Real-time PCR Master Mix (Toyobo) on StepOnePlus Real Time PCR System (ABI). 10-Fold diluted cDNA (2.5 μl) was add to the qRT-PCR mixture (12.5 μl SYBR Green Real-time PCR Master Mix (2x), 2 μl each forward and reverse primer (10 μm), 6 μl of double-distilled water). The cycling conditions in the qRT-PCR were 95 °C for 10 min, followed by 40 cycles of 95 °C for 15 s, 60 °C for 15 s, and 72 °C for 60 s. The primer sets for the segment 6 qRT-PCR can be found in the previous report (47). The nucleotide sequences of the forward and reverse primers for 18S rRNA were 5′-CGG ACA GGA TTG ACA GAT TG-3′ and 5′-CAA ATC GCT CCA CCA ACT AA-3′, respectively. The levels of vRNA, cRNA, and mRNA were normalized to 18 S rRNA (48).

##### Reverse Genetics to Produce Recombinant Viruses

Plasmid-based reverse genetics for viral generation was performed as previously described (49). Briefly, 8 PolI plasmids, in which viral RNA from strain A/WSN/33 (H1N1) was under the control of the human RNA polymerase I promoter and the mouse RNA polymerase I terminator, together with pCA-PB2, -PB1, -PA, and -NP, which express each viral protein derived from an influenza A virus strain A/WSN/33 (H1N1) (50), were transfected into 293T cells using Trans-IT 293 (Mirus). At 48 h post-transfection, culture supernatants were harvested and inoculated to MDCK cells for viral propagation. The titers of the stock viruses were determined using plaque assays in MDCK cells.

##### Minigenome Assay

Minigenome assay based on the dual-luciferase system was performed as previously described (51, 52). Briefly, A549 cells were transfected with pCA-NP, -PA, -PB1, and -PB2 or its mutants (0.2 μg each), pPolI/NP(0)Fluc(0) (0.2 μg), which express reporter vRNA encoding firefly luciferase gene, and pRL-null (Promega, 0.2 μg), which express *Renilla* luciferase as an internal control. The luciferase activity in the transfected A549 cells was measured using the Dual-Glo Luciferase Assay System (Promega) at 24 h post-transfection. Polymerase activity is calculated by normalizing the firefly luciferase activity to the *Renilla* luciferase activity. Polymerase activity of wild-type was set to 100%.

##### Growth Kinetics of Viruses in A549 Cells

A549 cells were infected with the indicated viruses at a multiplicity of infection of 0.001. After incubation at 37 °C for 1 h, the viral inoculum was replaced with DMEM supplemented with 1% BSA and 0.5 μg/ml of TPCK-treated trypsin, followed by further incubation at 37 °C. Culture supernatants collected at 12, 24, 48, 72, and 96 h post-infection were subjected to virus titration by plaque assays in MDCK cells.

## RESULTS

### 

#### 

##### Similarity between the Tertiary Structure of the PB2 Cap-binding Domain and Prokaryotic Acetyltransferases

**FIGURE 1.**
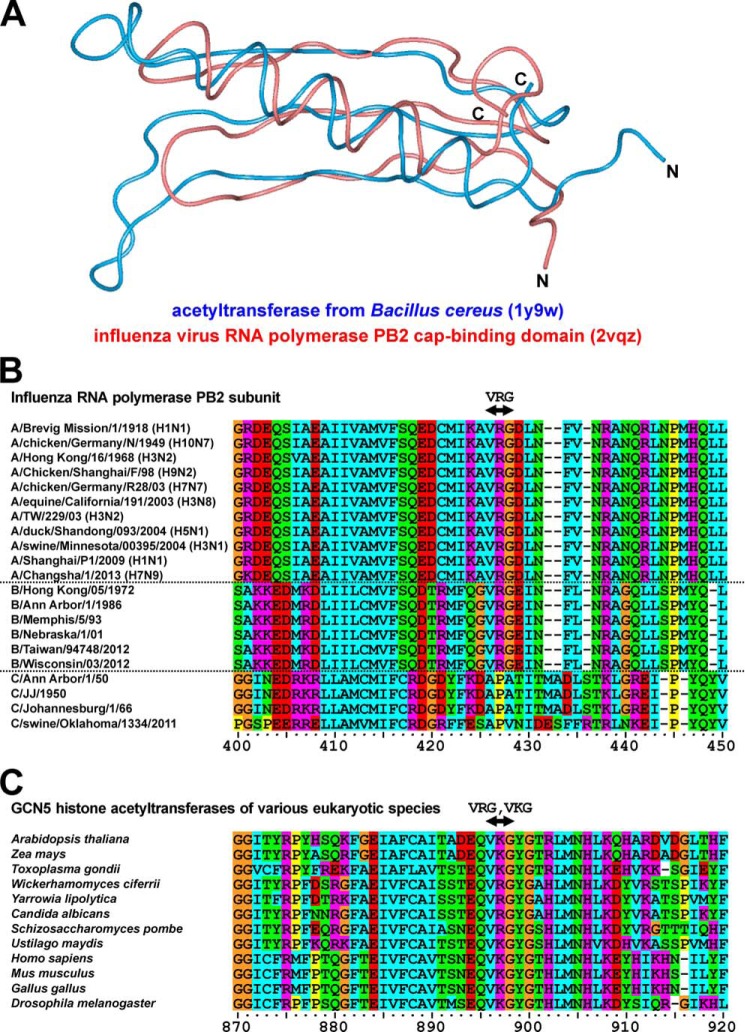
**Structural homology between PB2 and eukaryotic acetyltransferases.** *A,* similarity of tertiary structures between the cap-binding domain of the PB2 subunit from the influenza A virus (the *red ribbon*; PDB code 2vqz) and that of the acetyltransferase from bacterium *B. cereus* (the *blue ribbon*; PDB code 1y9w). *B,* amino acid alignment of cap-binding domains of PB2 across various strains of influenza viruses. Sequences were placed in the order of years when each strain had emerged. Groups of influenza A, B, and C viruses were separated by *dotted lines*. The VRG site was highly conserved in strains of influenza A and B viruses. This site was not present in any strains of influenza C viruses. *C,* amino acid alignment of GCN5 acetyltransferases across various eukaryotic species. The VRG site and its corresponding sequence, the VKG site, are conserved in various GCN5 eukaryotic acetyltransferases. These amino acid sequences were aligned in a multiple-sequence alignment program, ClustalX. *Orange*, glycine; *yellow*, proline; *cyan*, hydrophobic neutral amino acids; *green*, hydrophilic neutral amino acids; *red*, acidic amino acids; *magenta*, basic amino acids.

In this study, we investigated the central region of the PB2 subunit, which includes the cap-binding domain, and searched for proteins containing a similar tertiary structure using the MATRAS evolution program to compare the three-dimensional structures of proteins (42). Using this approach, we identified many types of prokaryotic acetyltransferases (27 of the 87 molecules shown in supplemental Table S1). Comparison of the tertiary structures of the PB2 cap-binding domain (PDB code 2vqz; amino acids 318 to 483) and acetyltransferase of *Bacillus cereus* (PDB code 1y9w) revealed similarities in the Cα-traces of loops, α-helices, and β-sheets (Fig. 1*A*). In addition, we investigated functional domains involved in acetyltransferase activity of the cap-binding domain of PB2. Amino acid sequences around this domain were almost completely conserved across the various strains of influenza A viruses from 1918 to 2013 and influenza B viruses from 1972 to 2012; the VRG site was conserved in all strains of influenza A and B viruses (Fig. 1*B*). However, the VRG site was not found in any strains of influenza C viruses (Fig. 1*B*). This functional domain (VRG and its corresponding sequence, VKG) is widely conserved in eukaryotic GCN5 acetyltransferases (Fig. 1*C*), and is essential for the interaction between acetyltransferase and its ligand, acetyl-CoA (22). Therefore, the results derived from these structural analyses suggest that the cap-binding domain of PB2 interacts with acetyl-CoA and exhibits acetyltransferase activity.

##### Interaction of the PB2 Cap-binding Domain with Acetyl-CoA

**FIGURE 2.**
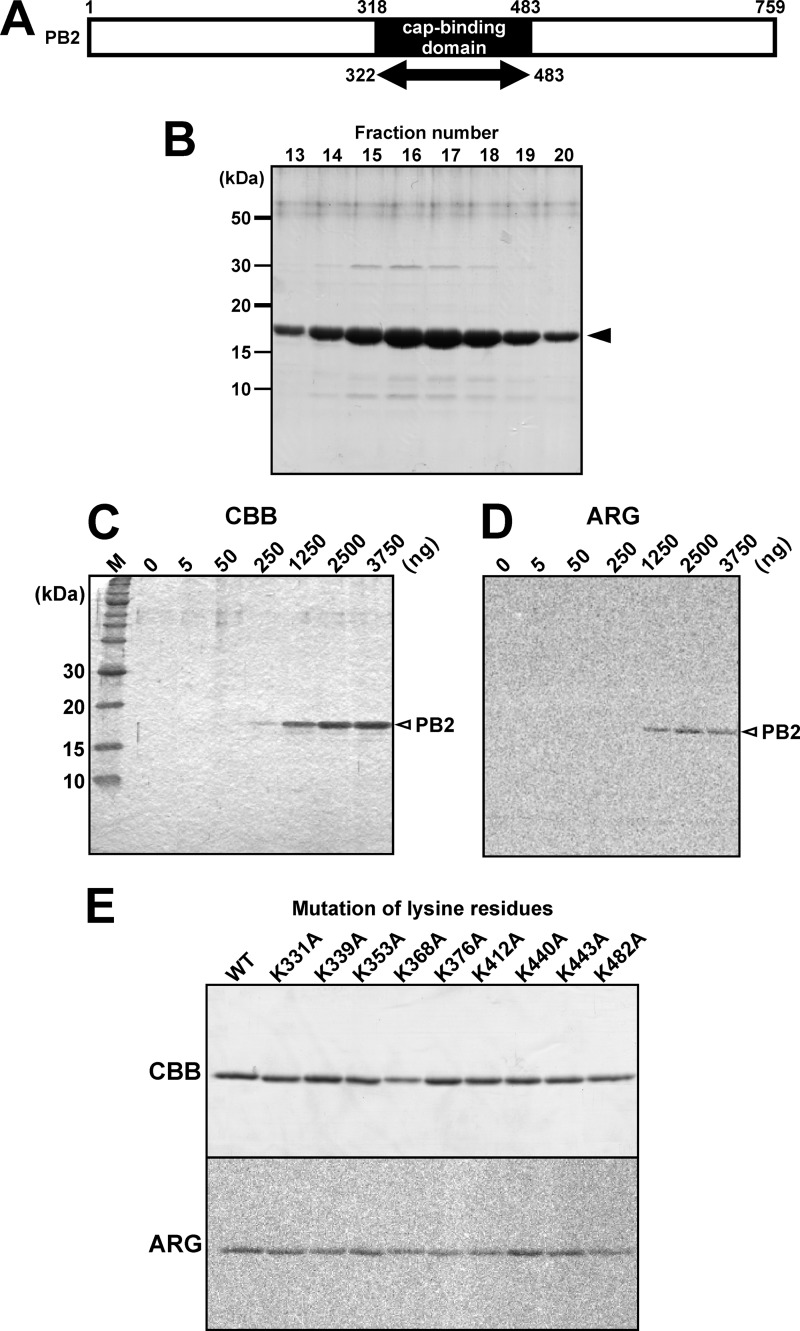
**Interaction of the PB2 cap-binding domain with acetyl-CoA.** *A,* diagram of the PB2 subunit protein. A partial region of PB2 containing the cap-binding domain (amino acids 322–483), whose molecular mass was calculated at ∼18.5 kDa, was expressed in *E. coli* to perform following biochemical analyses. *B,* the bands of recombinant PB2 purified by cation-exchange chromatography were detected in fractions 13–20 (*arrowhead*). *C,* after incubation with [^14^C]acetyl-CoA, different amounts of the recombinant PB2 were separated in a 14% SDS-PAGE gel. *D,* autoradiography of the Coomassie Brilliant Blue-stained gel shown in *A*. The PB2 cap-binding domain signal was observed in the absence of histone subunits. *E,* point mutation of each lysine residue to alanine did not eliminate the signal on the PB2 cap-binding domain. *CBB*, Coomassie Brilliant Blue staining; *ARG*, autoradiography.

The region of the recombinant protein was from amino acids 318 to 483, which covers the cap-binding domain (Fig. 2*A*). The molecular mass of this recombinant protein was calculated at ∼18.5 kDa. To perform biochemical investigation of the PB2, a partial region of PB2 containing the cap-binding domain (amino acids 322–483; GenBank Accession number AAO46493) was expressed in the BL21 strain of *E. coli*. The recombinant PB2 tagged with His_6_ and roughly purified with nickel-agarose resin was further purified by cation-exchange chromatography (Fig. 2*B*). Using the recombinant cap-binding domain (amino acids 322–483; Fig. 2*A*) of PB2, we performed biochemical analyses with [^14^C]acetyl-CoA to investigate whether this partial recombinant protein exhibited acetyltransferase activity. A positive signal of recombinant partial PB2 was detected by incubation with only PB2 cap-binding domain (Fig. 2, *C* and *D*), suggesting two possibilities. (i) Radioactive isotopes [^14^C] were present in acetyl groups of acetyl-CoA. Therefore, the first possibility was that this acetyl group might be transferred to the PB2 cap-binding domain itself, suggesting that this domain possessed an autoacetylation activity. (ii) The second possibility was that the PB2 cap-binding domain might interact with [^14^C]acetyl-CoA. The target amino acid residue for acetylation is generally lysine (22), and 9 lysine residues were contained in this recombinant PB2 cap-binding domain. To test the autoacetylation activity of the PB2 cap-binding domain, each lysine residue was substituted with an alanine, after which the acetylation level of mutant recombinant PB2 was measured using [^14^C]-labeled acetyl-CoA (Fig. 2*E*). Interestingly, the mutations did not reduce the radioactive signals and, therefore, the PB2 cap-binding domain is likely to lack an autoacetyltransferase activity but bind to acetyl-CoA. In these experiments, the positive signals remained in the PB2 recombinant protein even after boiling, suggesting that the PB2 recombinant protein bound to acetyl-CoA via covalent bonds. Accordingly, we next tested whether the PB2 cap-binding domain interacts with acetyl-CoA.

**FIGURE 3.**
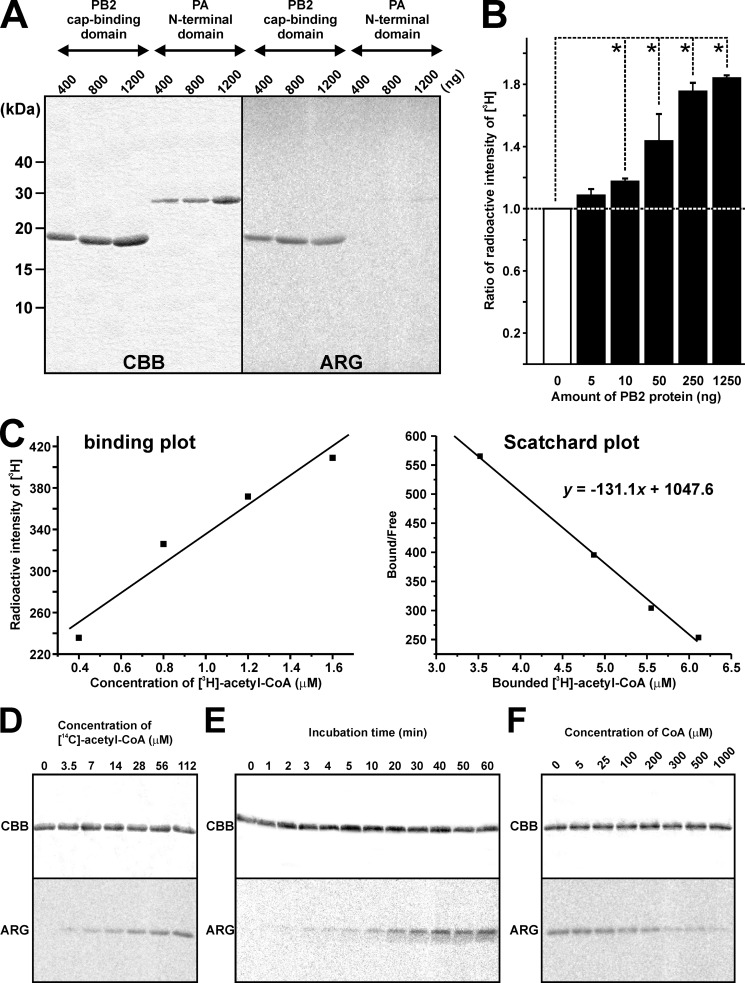
**Biochemical characterization of the interaction between the PB2 cap-binding domain and acetyl-CoA.** *A,* recombinant proteins of the PB2 cap-binding domain and the PA N-terminal domain were incubated with [^14^C]acetyl-CoA in parallel, and the positive signals were detected only in PB2 bands. *B,* the ratio of the radioactive intensity of [^3^H] increased depending on the amount of recombinant PB2. Differences were analyzed for statistical significance by one-way analysis of variance, *n* = 3 or 4. *Error bars* = S.E. *, *p* < 0.05. *C,* Scatchard plot (*right panel*), which was calculated from the simple binding plot (*left panel*), demonstrated *K_d_* value of 131 μm. *D,* the same amount of recombinant PB2 was applied in all lanes (*upper panel*). Autoradiography indicates that the level of PB2 interacting activity was dependent on [^14^C]acetyl-CoA concentrations (*lower panel*). *E,* a long duration of incubation increased the interaction level of the recombinant PB2. *F,* a concentration of CoA (100 μm) equal to that of [^14^C]acetyl-CoA slightly inhibited interaction of the recombinant PB2. *CBB*, Coomassie Brilliant Blue staining; *ARG*, autoradiography.

A series of biochemical experiments were performed to understand the interacting activity of the PB2 cap-binding domain with acetyl-CoA in detail. To confirm that this ability was specific to PB2, recombinant proteins of the PB2 cap-binding domain and the PA N-terminal domain were incubated with [^14^C]acetyl-CoA at the same time. The positive signal was only detected in the bands of PB2, suggesting that acetyl-CoA specifically interacted with PB2 (Fig. 3*A*). Incubation of serial concentrations of the PB2 cap-binding domain with [^3^H]acetyl-CoA (1.2 μm; 233 mCi/mmol) for 30 min at 30 °C revealed that the ratio of radioactive intensity increased depending on the amount of recombinant PB2 protein (Fig. 3*B*). The kinetics of the interaction between the PB2 cap-binding domain and acetyl-CoA were investigated by tracing Scatchard plots based on the radioactive intensity of [^3^H], and the *K_d_* value was estimated to be ∼7.6 μm (Fig. 3*C*). This suggests that the high affinity between the PB2 cap-binding domain and acetyl-CoA prevented the complex from separating by SDS-PAGE shown in Fig. 2. A fixed amount of recombinant partial PB2 cap-binding domain was incubated with different concentrations of [^14^C]acetyl-CoA. We found that the intensity of the signal on partial PB2 was increased depending on the concentration of [^14^C]acetyl-CoA (Fig. 3*D*). In addition, a longer incubation with a fixed amount of [^14^C]acetyl-CoA enhanced the PB2 cap-binding domain signal, the intensity of which plateaued at 40 min, indicating that interactions between the cap-binding domain and acetyl-coA reached a saturation point (Fig. 3*E*). Notably, CoA can function as a competitor to acetyl-CoA in its interaction with HATs (36). Therefore, we subsequently incubated a fixed amount of the PB2 cap-binding domain and [^14^C]acetyl-CoA (112 μm) with varying concentrations of CoA. The reduction in the signal intensity of the PB2 cap-binding domain was observed, depending on the CoA concentration. Furthermore, incubation with 100 μm or more of CoA, concentrations equal to or higher than the [^14^C]acetyl-CoA concentration, attenuated the interaction with the PB2 cap-binding domain (Fig. 3*F*).

##### HAT Inhibitors Blocked the Acetyl-CoA-binding Activity of PB2 and Had Anti-influenza Effects

**FIGURE 4.**
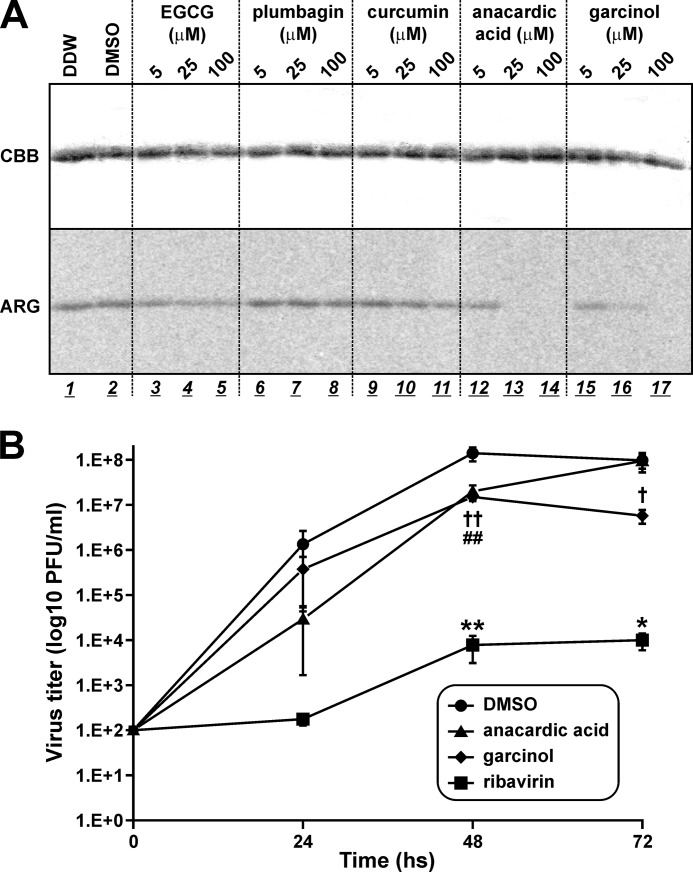
**Inhibition of the binding activity of recombinant PB2 with acetyl-CoA by HAT blockers.** *A,* HAT inhibitors were mixed in the reaction solution prior to incubation. Following addition of 1 mg of recombinant PB2 cap-binding domain proteins, incubation was performed for 30 min at 30 °C. Anacardic acid and garcinol showed strong inhibition of the binding activity of recombinant PB2 with [^14^C]acetyl-CoA. *CBB*, Coomassie Brilliant Blue staining; *ARG*, autoradiography. *B,* anti-influenza activity of HAT inhibitors was examined by means of a plaque assay using cultured MDCK cells. DMSO and ribavirin were used as negative and positive controls, respectively. The data are shown as mean ± S.E. (*n* = 4) and are representative of 3 independent experiments. Differences between the effect of each drug and the effect of DMSO were analyzed for statistical significance using analysis of variance. *, *p* < 0.05 and **, *p* < 0.01; DMSO *versus* ribavirin. ##, *p* < 0.01; DMSO *versus* anacardic acid. †, *p* < 0.05 and ††, *p* < 0.01; DMSO *versus* garcinol.

One HAT, p300/cAMP-response element-binding protein-associated factor, contains a Val/Lys/Gly (VKG) site that corresponds to the VRG site in GCN5 and the PB2 cap-binding domain, which is required for acetyl-CoA interaction (53). Results from a recent study, in which molecular docking simulation was used, suggest that anacardic acid competes with acetyl-CoA for the VKG site in p300/cAMP-response element-binding protein-associated factor (54). Analogues of anacardic acid are also known to target the acetyl-CoA binding site of other HATs, such as Tip60 (55). In addition to anacardic acid and its derivatives (56, 57), several types of natural chemicals have been reported to bind HATs, such as epigallocatechin-3-gallate (58), plumbagin (59), curcumin (60), and garcinol (61). Therefore, we investigated whether these chemicals can inhibit the interaction between the PB2 cap-binding domain and acetyl-CoA (Fig. 4*A*). Low concentrations (5 μm) of epigallocatechin-3-gallate slightly reduced the interaction between the PB2 cap-binding domain and acetyl-CoA, increasing the epigallocatechin-3-gallate concentration to 25 and 100 μm and resulted in similar, but not greater, levels of inhibition as observed with the 5 μm treatment. Plumbagin and curcumin at concentrations of 5 and 25 μm did not block the interaction, although concentrations of 100 μm did result in a slight attenuation of interaction activity. In contrast, anacardic acid and garcinol drastically inhibited the interaction, and concentrations of anacardic acid greater than 25 μm completely eliminated interaction, whereas only faint activity was detected by adding 25 μm garcinol; however, no interaction was detected with the addition of 100 μm garcinol. These results demonstrated that several HAT inhibitors also blocked the interaction between the PB2 cap-binding domain and acetyl-CoA.

Next, we examined the possibility of anti-influenza activity in these HAT inhibitors using a plaque assay (Fig. 4*B*). MDCK cells were infected with influenza A/PR/8/34 virus at a multiplicity of infection of 0.001 and treated with 0.5% of DMSO and 50 μm ribavirin, anacardic acid, or garcinol. Ribavirin was previously reported to have sufficient anti-influenza effects (62). Viral titers in supernatants were determined using influenza A NP immunostaining of treated MDCK cells. Viral titers were reduced by treatment with anacardic acid or garcinol for 48 h (DMSO *versus* anacardic acid, DMSO *versus* garcinol: *p* < 0.01), whose effects were weaker than that of ribavirin (DMSO *versus* ribavirin; *p* < 0.01). After treatment for 72 h, antiviral activity of anacardic acid was lost, but garcinol was maintained (DMSO *versus* garcinol; *p* < 0.05). These results suggested that binding of PB2 to acetyl-CoA is essential for viral replication and that HAT inhibitors (which block this activity) are useful as novel anti-influenza agents.

##### A Shared Location of the Binding Pocket for Acetyl-CoA and m^7^GTP

**FIGURE 5.**
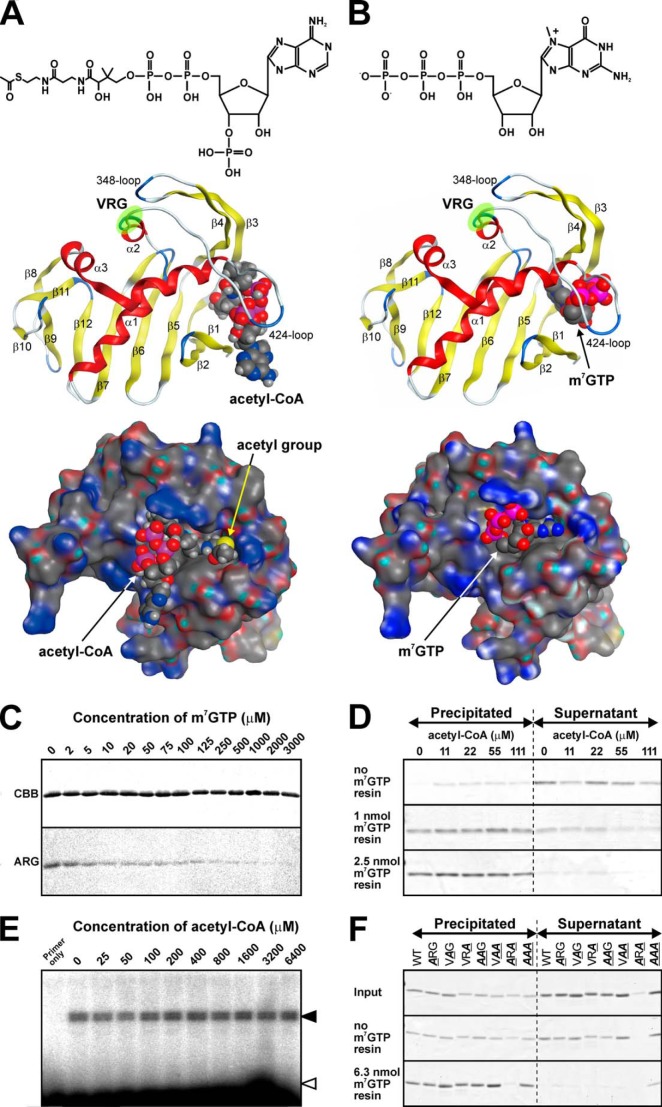
**Acetyl-CoA and m^7^GTP share the same PB2-binding pocket.** *A* and *B, upper panel* shows structural comparison between acetyl-CoA and m^7^GTP. Both molecules were equally constructed by series of a purine base, a ribose, and phosphates. *Middle* (ribbon structures) and *lower* (surface structures) panels shows results of binding simulation using the Molecular Operating Environment (MOE) software between the PB2 cap-binding domain and acetyl-CoA or m^7^GTP, respectively. The structure of the PB2 cap-binding domain with a bound m^7^GTP was based on data from the Protein Data Bank (PDB code 2vqz). The positions of the VRG sites are highlighted by *green circles. C,* 1 μg of recombinant PB2 cap-binding domain proteins was incubated with each concentration of m^7^GTP for 30 min at 30 °C. Enhancement of m^7^GTP concentrations inhibits the interaction between PB2 and acetyl-CoA. *CBB*, Coomassie Brilliant Blue staining; *ARG*, autoradiography. *D,* the pull-down assay using m^7^GTP immobilized on an agarose resin showed that acetyl-CoA did not inhibit the binding of recombinant PB2 to m^7^GTP. The recombinant PB2 remained in the supernatant and was partly precipitated without the m^7^GTP-resin. It was trapped in 1 or 2.5 nmol of m^7^GTP-resin independently of different concentrations of acetyl-CoA. *E,* the *in vitro* transcription assay. Neuraminidase gene-specific primer extension showed that even an excess amount of acetyl-CoA did not affect the transcriptional activity of purified RNP. *Filled* and *open arrowheads* show the double bands of mRNA-specific products and unbound primers, respectively. *F,* amino acid substitutions at the VRG site did not affect the binding to m^7^GTP. “Input” shows the total amount of each recombinant protein, and supernatants after precentrifugation were used in these experiments. Only the **A**R**A** mutant protein could not remain in the supernatant after pre-centrifugation and was not useful for this assay. Any recombinant proteins could remain in supernatants after incubation without the m^7^GTP-resin. They bound to the m^7^GTP-resin and were precipitated, and the **AAA** mutant protein partially remained in the supernatant even in the presence of the resin.

To identify the binding pocket of acetyl-CoA in the PB2 cap-binding domain, we performed a binding simulation using the MOE software. This simulation suggested that acetyl-CoA was trapped in the 424-loop (Fig. 5*A*). Additionally, the VRG site, a putative consensus site for interaction with acetyl-CoA, did not directly bind to acetyl-CoA in PB2, but was located at the basal part of the 424-loop. Thus, this site may function to maintain the proper tertiary structures, which contained a binding pocket for acetyl-CoA. This binding pattern was different from that of a eukaryotic GCN5 acetyltransferase, which was reported to directly interact with acetyl-CoA via the VRG site (40).

A previous report showed that PB2 binds to and sequesters the cap structure of host mRNA, and that the tertiary structure of the PB2 cap-binding domain, whereas it was bound to m^7^GTP has since been resolved (16). Then, we compared the binding positions between acetyl-CoA and m^7^GTP. These binding simulations suggested that the active pockets of these ligands were closely located or even shared similar segments of the protein (Fig. 5, *A* and *B*). Thus, we anticipated that excess concentrations of m^7^GTP would inhibit the binding of acetyl-CoA to the active pocket, whereas also preventing the chemical interaction. To demonstrate it, PB2 and [^14^C]acetyl-CoA were incubated in the presence of different concentrations of m^7^GTP. The interaction with acetyl-CoA was inhibited depending on the m^7^GTP concentration (Fig. 5*C*). Therefore, we demonstrated that the cap-binding domain of PB2 possesses a dual function: binding to m^7^GTP and interaction with acetyl-CoA. Next, we examined the possibility that an excess concentration of acetyl-CoA could compete with m^7^GTP for the binding site (Fig. 5*D*). Contrary to our expectations, acetyl-CoA did not inhibit the binding of recombinant PB2 to m^7^GTP immobilized on the agarose resin, and the recombinant PB2 remained in the supernatant and was partly precipitated without the m^7^GTP resin; these data suggested that recombinant PB2 was partially aggregated as a result of the rotation (Fig. 5*D*). Nonetheless, it was trapped in the m^7^GTP resin independently of acetyl-CoA (Fig. 5*D*). To examine the possibility that acetyl-CoA can affect RNA synthesis of RdRp, we performed an *in vitro* transcription assay using purified RNP and analyzed the transcripts by means of the neuraminidase gene-specific primer extension method (Fig. 5*E*). Viral mRNA was synthesized by incubation of purified RNP and globin mRNA as a donor of m^7^GTP in the presence of different concentrations of acetyl-CoA. Even an excess concentration of acetyl-CoA did not influence transcriptional activity of purified RNP (Fig. 5*E*). Considering the results of the competitive binding experiments between acetyl-CoA and m^7^GTP (Fig. 5, *C* and *D*), it appears that the binding affinity for m^7^GTP is much higher than that for acetyl-CoA. To elucidate the effect of the VRG site on the binding of m^7^GTP, we produced recombinant versions of the PB2 cap-binding proteins, whose amino acid residues in the VRG site were mutated; we then incubated each of these proteins with immobilized γ-aminophenyl-m^7^GTP (Fig. 5*F*). A precipitate formed during preincubation was removed by centrifugation, and only the supernatant was used for this assay. After this centrifugation, the **A**R**A** mutant was precipitated, and amount of the protein remaining in the supernatant was too small for further analysis using the cap-resin pull-down assay (Fig. 5*F*). The VRG site is located at the root of the 424-loop (*middle panels* of Fig. 5, *A* and *B*), and this pattern of mutation of the amino acid sequence may critically affect the position of the 424-loop and maintenance of the correct tertiary structure. Although the band of the **A**R**A** mutant in the precipitate was almost undetectable, the band in the supernatant was also less intense compared with the other recombinant proteins, suggesting that this mutant protein was degraded during preincubation (Fig. 5*F*). Wild-type and mutant recombinant proteins of the PB2 cap-binding domain remained in the supernatants without m^7^GTP-resin. Although the **AAA** mutant partly remained in the supernatant, wild-type, **A**RG, V**A**G, VR**A**, **AA**G, and V**AA** mutant proteins were completely bound to the m^7^GTP-resin and precipitated (Fig. 5*F*). These results showed that the VRG site was not involved in the binding of PB2 to m^7^GTP.

##### The VRG Site Is Essential for Interaction with Acetyl-CoA, RNA Polymerase Activity, and Viral Growth

**FIGURE 6.**
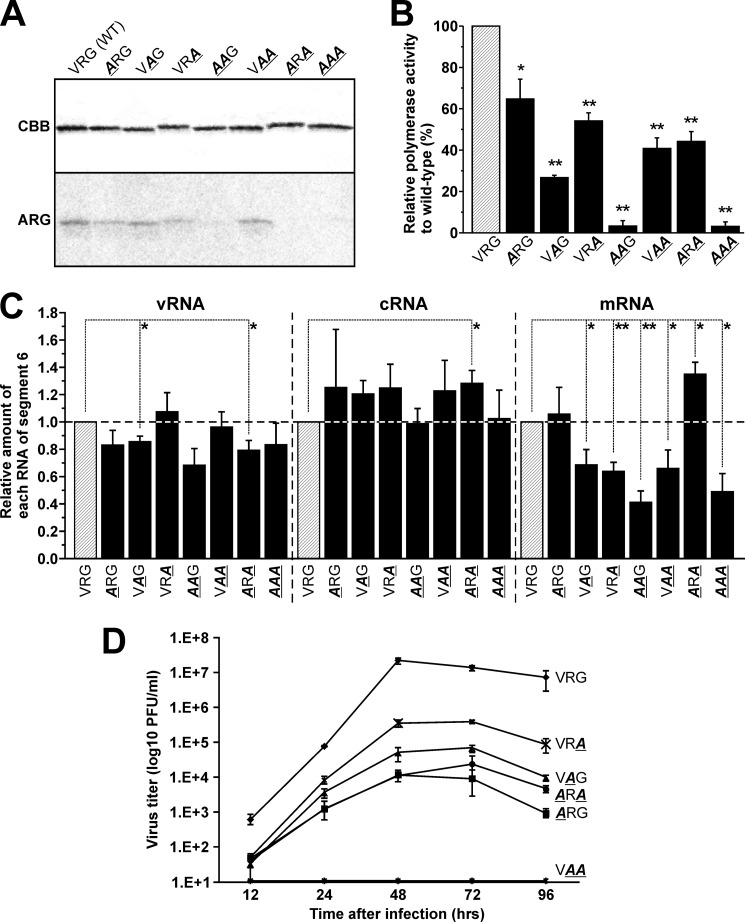
**The VRG in the PB2 subunit is essential for interaction with acetyl-CoA, RNA polymerase activity, and viral growth.** *A,* substitution of amino acid residues at the VRG site with one or more alanine residues altered interaction activity. The amount of recombinant PB2 was fixed at 1 μg in all experiments. *B,* minigenome assay to investigate effects on polymerase activity of viral RdRp by amino acid mutation in the VRG site. Differences were analyzed for statistical significance by one-way analysis of variance. *n* = 3, *error bars* = S.E. *, *p* < 0.05 and *, *p* < 0.01, respectively. *C,* strand-specific qRT-PCR for measuring a relative amount of vRNA, cRNA, and mRNA of segment 6. The level of mRNA of each mutant, except for **A**RG, was significantly decreased. *, *p* < 0.05 and **, *p* < 0.01. *D,* time course of titers from viruses generated from stock mutant viruses.

A binding simulation between the PB2 cap-binding domain and acetyl-CoA suggested that the VRG site played a crucial role in maintaining the tertiary structure of the acetyl-CoA binding site (Fig. 5*A*). We decided to elucidate the function of the VRG site in relationship to the binding of PB2 to acetyl-CoA, to its other biochemical function, and to viral replication. Accordingly, we produced a recombinant protein and a recombinant virus containing amino acid mutations in the VRG site and assessed the effects on its (*a*) interaction with acetyl-CoA, (*b*) RNA polymerase activity, and (*c*) viral replication. First, the mutant recombinant partial PB2, in which the valine, arginine, and glycine residues were substituted with alanine, was expressed in *E. coli*; any changes in their interaction levels were measured using [^14^C]acetyl-CoA (Fig. 6*A*). Substitution of a single valine or glycine residue (**A**RG or VR**A**) slightly reduced the interaction level of partial PB2, whereas a single substitution of arginine (V**A**G) did not affect interaction. A double substitution of Val/Arg (**AA**G) and Val/Gly (**A**R**A**) eliminated the interaction between recombinant partial PB2 and acetyl-CoA, but no effect was observed with the combined Arg/Gly (V**AA**) substitution. A triple substitution of all three amino acid residues (Val/Arg/Gly to **AAA**) also eliminated any interaction. A combined mutation of valine and other amino acid residues at the VRG site eliminated any enzymatic activity, thus indicating the importance of the valine residues in the interaction.

Next, to understand the importance of the interaction between PB2 and acetyl-CoA with regard to polymerase activity, we performed a minigenome assay and discovered changes in polymerase activity that were dependent on point mutations at the VRG site. Notably, two mutations (**AA**G and **AAA**) that eliminate any PB2 interaction with acetyl-CoA were found to drastically reduce transcription (Fig. 6*B*). In the **A**R**A** mutation, however, the polymerase activity was significantly reduced but maintained despite eliminating the acetyl-CoA binding activity, which was similar to the results of **AA**G and **AAA** mutations (Fig. 6, *A* and *B*). These results suggested that the binding site of acetyl-CoA was lost and that the binding site of m^7^GTP was spared by the **A**R**A** mutation. Relative polymerase activity was significantly attenuated with other mutations in the VRG site (Fig. 6*B*).

To examine which strands of RNA of influenza virus were down-regulated by mutations in the VRG site, we performed strand-specific qRT-PCR (Fig. 6*C*). Cultured A549 cells were transfected with pCA-NP, PA, PB1, and PB2 or its mutants, and with pPolI-NA (segment 6). Although small alterations of vRNA and cRNA levels were caused by substitutions of amino acid residues in the VRG site, the mRNA level largely decreased, except for the **A**R**A** mutation (Fig. 6*C*). In particular, the **AA**G and **AAA** mutations, which drastically decreased total RNA levels in a minigenome assay (Fig. 6*B*), showed the largest reduction of mRNA levels among 7 mutation patterns and no changes in cRNA levels. There was a tendency for increasing levels except for these mutation patterns, suggesting that mutations in the VRG site affect the RNA polymerase activity related to transcription. The **A**R**A** mutation decreased the total RNA level in a minigenome assay (Fig. 6*B*). On the other hand, strand-specific qRT-PCR showed that this mutation decreased the vRNA level, but increased cRNA and mRNA levels (Fig. 6*C*). A decrease of the vRNA level might reflect the result of reduction of the total RNA level by the **A**R**A** mutation. Amino acid mutations in the VRG site had different effects on cRNA and mRNA levels (Fig. 6*C*). Previous reports showed that molecular mechanisms of synthesis of cRNA and mRNA in influenza A virus are different (63, 64), and the binding activity of PB2 to acetyl-CoA might specifically participate in mRNA synthesis.

Finally, we investigated the effect of the VRG site amino acid sequence mutations on viral growth. In this experiment, stock viruses were first produced by inoculating MDCK cells with the supernatant of transfected 293T cells, after which the viral titer of stock viruses was measured in the MDCK cells. In the case of **AA**G and **AAA** mutations, the titer of stock viruses was less than 10 pfu/ml, which was too low a concentration to quantify viral growth (Table 1). These results indicate that inhibition of polymerase activity related to transcription prevented replication of the virus. Titers of stock viruses containing other mutants were greater than 10^4^ pfu/ml, and were therefore used for the evaluation of viral growth by infection of A549 cells. The viral titer with V**AA** mutations failed to grow (Fig. 6*D*). The **A**RG, V**A**G, VR**A**, and **A**R**A** mutations also inhibited viral growth. Therefore, these data indicate that the VRG site in the PB2 cap-binding domain is essential for polymerase activity of viral RNA polymerase and subsequent viral growth due to the interaction between the PB2 and an acetyl-CoA.

**TABLE 1:** **Titers of stock viruses in the infection experiment (Fig. 6*D*)**

Virus	Titer
	*pfu/ml*
VRG (WT)	3.7 × 10^8^
**A**RG	7.8 × 10^6^
V**A**G	7.6 × 10^5^
VR**A**	3.0 × 10^7^
**AA**G	<10
V**AA**	8.0 × 10^4^
**A**R**A**	7.2 × 10^6^
**AAA**	<10

## DISCUSSION

In this study, bioinformatics analyses revealed that the tertiary structure of the cap-binding domain of PB2 was similar to that of bacterial acetyltransferases and that the VRG site, which is present in many acetyltransferases across multiple species, was also conserved in PB2. This suggests that the cap-binding domain performs dual functions in that it interacts with both the RNA cap and acetyl-CoA. However, through the use of biochemical approaches, we obtained data that indicates the PB2 cap-binding domain does not possess acetylation activity, but interacts only with acetyl-CoA. This interaction could be significantly blocked with CoA, HAT inhibitors, and m^7^GTP. The VRG site is thought to be required for RNA polymerase activity of viral RdRp as well as viral growth.

What is the significance of the interaction between the PB2 cap-binding domain and acetyl-CoA? Our data indicate that amino acid sequence mutations in the VRG site (**AA**G and **AAA**) that eliminate any interaction between the PB2 and an acetyl-CoA (Fig. 6*A*) also result in the loss of polymerase activity of viral RdRp (Fig. 6, *B* and *C*) and of viral replication (Fig. 6*D*). Previous reports have shown that acetyl-CoA stimulates RNA polymerase II transcription as well as promoter binding by increasing its affinity for TFIID (38). Taken together, the interaction of the PB2 with a molecule of acetyl-CoA is believed to regulate the transcription of viral RdRp via PB2. However, the possibility that PB2 exhibits some acetyltransferase activity cannot be completely ruled out. Although in this study, single lysine mutations in the PB2 cap-binding domain failed to eliminate acetyltransferase activity (Fig. 2*E*), autoacetylation may occur on the other lysine residues during the monitoring period. To determine whether autoacetylation occurs, we attempted to produce various mutant PB2 cap-binding domains in which each of the 9 lysine residues were substituted with alanine residues. Unfortunately, the resulting mutant recombinant protein could not be solubilized and, therefore, we could not investigate this hypothesis using biochemical analysis. The recombinant protein used in this report contained only the PB2 cap-binding domain, and it is possible that the catalytic domain required for acetylation is located in other parts of the PB2 protein or in one of the other subunits that comprise the viral RdRp. In addition, sense mutations in the VRG site did not influence the binding affinity of PB2 for m^7^GTP (Fig. 5*F*). These results suggested that the VRG sequence in PB2 was not used for direct interaction with acetyl-CoA, but played a crucial role in the docking with acetyl-CoA, not with m^7^GTP.

The docking simulation performed here suggested that acetyl-CoA shares the same active pocket as m^7^GTP and, consequently, m^7^GTP can dose dependently inhibit the interaction of recombinant PB2 with acetyl-CoA (Fig. 5). All amino acid residues involved in the interaction with m^7^GTP have previously been identified and, notably, analyses of these residues did not include the VRG site (17). These results suggest that the amino acid residues necessary for interacting with each ligand are localized on the same surface of the binding pocket, but are able to distinguish between ligands, acetyl-CoA and m^7^GTP, based on their structural differences (Fig. 5, *A* and *B*). These binding pockets may functionally interact with each other to enhance the affinity of other ligands to the PB2 protein during the viral life cycle. Although the interaction with acetyl-CoA is inhibited depending on the m^7^GTP concentration (Fig. 5*C*), the excess amount of acetyl-CoA does not kick m^7^GTP out of the recombinant PB2 (Fig. 5*D*). These results suggest that acetyl-CoA and m^7^GTP can share the same binding pocket, but this pocket selectively interacts with m^7^GTP with much higher affinity compared with acetyl-CoA. Accordingly, a high concentration of acetyl-CoA has no effect on the transcriptional activity of purified RNP *in vitro* (Fig. 5*E*). Thus, acetyl-CoA is not involved in RNA polymerase activity *in vitro*. On the other hand, amino acid sequence mutations in the VRG site, which is essential for binding with acetyl-CoA (Fig. 6*A*), attenuate the RNA polymerase activity (Fig. 6, *B* and *C*) and viral replication (Fig. 6*D*). To elucidate the function of this acetyl-CoA binding ability, the combined crystal structure of the PB2·acetyl-CoA complex should be analyzed in detail, and we are currently working on this strategy. Interestingly, the **A**R**A** mutation does prevent acetyl-CoA interaction and decreased vRNA level, but increases cRNA and mRNA levels (Fig. 6, *B* and *C*), suggesting that this mutation enhances transcriptional levels from vRNA to cRNA and mRNA independently of binding to acetyl-CoA. In contrast, the partial recombinant protein with this pattern of amino acid substitution is easily aggregated and precipitated *in vitro* (Fig. 5*F*). The whole amino acid sequence may support the maintenance of the correct tertiary structure and enhance its RNA synthesis activity.

The *K_d_* value of the interaction between the PB2 cap-binding domain and acetyl-CoA calculated by the Scatchard plot was estimated to be 131 μm (Fig. 3*C*). The cytoplasmic concentration of acetyl-CoA in mammalian cells varies from 1 to 50 μm (65, 66). These results suggest that PB2 does not have sufficient affinity for acetyl-CoA in infected cells. In contrast, the N-terminal region of PB2 was reported to possess a mitochondrial-targeting signal and, as predicted, both human and avian PB2 viruses were found to localize to the mitochondria of transfected cells (67). Acetyl-CoA is abundant in the mitochondria where the Krebs cycle and fatty acid synthesis occurs, and it is likely that PB2 may sequester the acetyl-CoA from these reactions to interact with it.

## Supplementary Material

Supplemental Data

## Footnotes

2 The abbreviations used are:

RdRp: RNA-dependent RNA polymerase
HAT: histone acetyltransferase
DMSO: dimethyl sulfoxide
MDCK: Madin-Darby canine kidney
TPCK: l-(tosylamido-2-phenyl)ethyl chloromethyl ketone
NP: nucleoprotein
RNP: ribonucleoprotein
qRT: quantitative RT
PDB: Protein Data Bank
vRNA: viral RNA.
