# Supplementary material for: A Novel Functional Site in the PB2 Subunit of Influenza A Virus Essential for Acetyl-CoA Interaction, RNA Polymerase Activity, and Viral Replication
Source: J Biol Chem. 2014 Jul 25;289(36):24980–94. doi: 10.1074/jbc.M114.559708 (PMC4155666; doi:10.1074/jbc.M114.559708)
Supplement: Supplemental Data [file supp_289_36_24980__index.html]

A Novel Functional Site in the PB2 Subunit of Influenza A Virus Essential for Acetyl-CoA Interaction, RNA Polymerase Activity, and Viral Replication — A Novel Functional Site in the PB2 Subunit of Influenza A Virus Essential for Acetyl-CoA Interaction, RNA Polymerase Activity, and Viral Replication — Novel Functional Site in Influenza RNA Polymerase — Supplemental Data 

# A Novel Functional Site in the PB2 Subunit of Influenza A Virus Essential for Acetyl-CoA Interaction, RNA Polymerase Activity, and Viral Replication

## Supplemental Data

**Files in this Data Supplement:**

- Table S1 (.xls, 35 KB) - Supplementary Table Table S1. The list of the tertiary structures similar to the cap-binding domain of PB2.
